# A high spatial resolution dataset for anthropogenic atmospheric mercury emissions in China during 1998–2014

**DOI:** 10.1038/s41597-022-01725-4

**Published:** 2022-10-06

**Authors:** Weicen Chang, Qiumeng Zhong, Sai Liang, Jianchuan Qi, Jetashree

**Affiliations:** 1 Key Laboratory for City Cluster Environmental Safety and Green Development of the Ministry of Education, School of Ecology, Environment and Resources, Guangdong University of Technology, Guangzhou, Guangdong 510006 P. R. China; 2 School of Environment, Beijing Normal University, Beijing, 100875 P. R. China

**Keywords:** Environmental sciences, Environmental social sciences

## Abstract

China is the largest atmospheric mercury (Hg) emitter globally, which has been substantially investigated. However, the estimation of national or regional Hg emissions in China is insufficient in supporting emission control, as the location of the sources may have significant impacts on the effects of Hg emissions. In this concern, high-spatial-resolution datasets of China’s Hg emissions are necessary for in-depth and accurate Hg-related studies and policymaking. Existing gridded datasets are constructed using population distribution as the proxy, which is limited as Hg emissions are closely related to energy consumption and economic processes. This study constructs a dataset of anthropogenic atmospheric Hg emissions in China gridded to a 1 km resolution during 1998–2014. This dataset is produced based on data of land uses, individual enterprises, roadmaps, and population, uncovering Hg emissions in agriculture, industries, services, and residents. This dataset can promote the reliability of Hg-related studies at a high spatial resolution. Moreover, this dataset can support spatially explicit Hg reduction of economic sectors.

## Abstract (summary)

**Table:** 

Measurement(s)	anthropogenic mercury emissions in China
Technology Type(s)	MATLAB, ArcGIS

## Background & Summary

Mercury (Hg) is a highly toxic substance^1^ threatening the ecosystems and human health on a global scale^2,3^. Human activity is a critical source of Hg emissions and releases. Approximately 30% of global atmospheric Hg emissions each year are anthropogenic^4–6^, and 60% of global Hg emissions are legacy emissions from anthropogenic sources. The remaining 10% are from natural sources^1,7^. China, the largest atmospheric Hg emitter globally^1^, is a major battlefield for implementing the Minamata Convention on Mercury. To support China’s Hg-control campaign, scholars have complied various inventories revealing the features and patterns of Hg emissions in China. Most inventories estimate Hg emissions by provinces and emission sources^8–12^, while some studies also focus on specific critical sources, including coal combustion^13,14^, nonferrous metal smelting^15,16^, biomass burning^17^, waste incineration^18^, etc.

However, as suggested by the Global Mercury Assessment (GMA) 2018^1^, the estimation of national or regional emissions is only the first step in Hg emission analysis. The location of the emission sources may have significant impacts on the transport and fate of Hg. For example, mountain ranges and weather patterns usually vary with different locations, which will affect the Hg transport. Thus, it is important to construct Hg emission inventories at fine spatial scales. To investigate the distribution of Hg emissions and the consequences, scholars have constructed multiple global high-spatial-resolution anthropogenic atmospheric Hg emission datasets. With the inventories compiled by the UNEP and Arctic Monitoring and Assessment Programme (AMAP), the grid maps of global Hg emissions^19–23^ are established by emission sources at the resolution of 0.5° × 0.5° and 0.25° × 0.25°. Moreover, the Emissions Database for Global Atmospheric Research (EDGAR) has published gridded global Hg emission maps (EDGARv4.tox1 and tox2). This dataset is generated by emission sources at the resolution of 0.1° × 0.1°^24,25^, covering 1970–2012. However, in these datasets, proxy data of point sources are relatively complete only in certain developed regions. In contrast, the proxy of population distribution is widely used to distribute emissions in other regions (China included), or even for some critical sources, limiting data reliability^19,24,25^. In this regard, to improve the data quality of gridded Hg emissions in China, scholars have developed datasets based on localized data for China. However, in these constructed datasets, Hg emissions are distributed with proxy data of point sources only in a few cases (e.g., large coal-fired power plants, metal smelting, and cement production), while the others are still in terms of area sources^8,10^. Besides, these datasets only cover data for limited time spans.

**Fig. 1:**
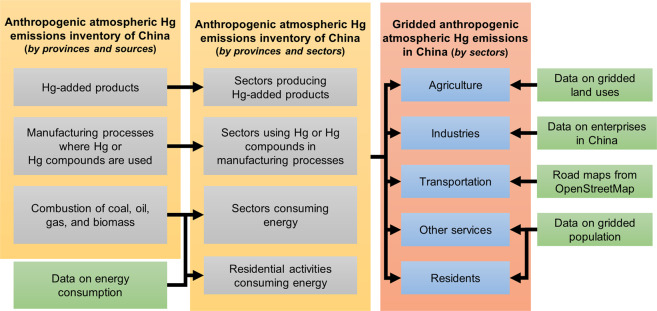
Methods used for constructing the high-spatial-resolution gridded anthropogenic atmospheric Hg emissions dataset for China.

To overcome these limitations and provide spatially explicit support for China’s Hg emission control efforts, this study constructed a high spatial resolution (1 km × 1 km) gridded dataset for anthropogenic atmospheric Hg emissions in China. This dataset contains grid maps of Hg emissions from 1998–2014 categorized by sectors in the economic system (Urban and rural residents are also treated as sectors in this study). Gridded estimates of the total Hg (THg) and of the three species, i.e., gaseous elemental Hg (Hg0), gaseous oxidized Hg (HgII), and particulate-bound Hg (Hg_P_), are given separately. The dataset is established with a top-down approach based on the schematic illustrated in Fig. 1. We use the anthropogenic atmospheric Hg emission inventory of China compiled by Wu *et al*.^11,12^, which provides the Hg emissions of China by provinces and emission sources. Then, we transform the inventory by provinces and sources into one by provinces and sectors. Finally, we use proxy data to distribute Hg emissions in sectors into grids. Gridded land use data is used to proxy Hg emissions in the agriculture sector. The Chinese Industrial Enterprises Database (CIED), in which the locations of enterprises are given, is used to distribute Hg emissions in industries. The road maps obtained from OpenStreetMap are used to distribute Hg emissions in the transportation sector, and the gridded population data is used as the proxy for emissions from other service sectors and residents.

With these improved proxies, this dataset can promote the reliability of Hg-related studies at a high spatial resolution from three perspectives. First, scholars can identify critical areas for Hg emissions, including primary levels of administrative regions, industrial zones, transportation junctions, populated areas, etc. With these identified hotspots, subsequent emission control measures can be more specific and targeted, rather than generalized. Second, this dataset can be used as an input to various models to simulate the transport and deposition of Hg, as well as exposure to Hg, therefore supporting studies concerning environmental impacts and human health risks. Moreover, this dataset can be integrated into global Hg emission grid maps to improve their data quality and support global-scale simulations. Last but not least, this dataset can support Hg reduction from the perspective of the economic system, by facilitating the identification of the top economic sectors driving Hg emissions in specific areas or zones during specific time periods.

## Methods

The construction procedure of China’s high-resolution spatial gridded anthropogenic atmospheric Hg emission dataset mainly consists of two parts. First, based on existing atmospheric Hg emission inventories for each of China’s provincial administrative regions and emission sources, we constructed an inventory for China’s provincial administrative regions by sectors^11,12^. Then, the atmospheric Hg emission inventories of each provincial administrative region and each sector in China were gridded with proxy data.

### Converting China’s provincial Hg emission inventory by sources to by sectors

There are 6 categories and 24 sources in the Hg emission inventory of Wu *et al*.^11,12^. We use the sectoral classification of the Carbon Emission Accounts & Datasets (CEADs) energy consumption database^26,27^ (Table 1). The use of Hg in processes is mapped to corresponding sectors. According to CEADs energy usage, emissions occurring due to the combustion of fuels are mapped to each sector. The matching relationship matrix is shown in Table S1.

**Table 1:** Sectoral classifications of the dataset.

Indexes	Sectors	Sub-sectors
1	Agriculture	Agriculture
2	Industries	Coal Mining and Dressing
3		Petroleum and Natural Gas Extraction
4		Ferrous Metals Mining and Dressing
5		Nonferrous Metals Mining and Dressing
6		Nonmetal Minerals Mining and Dressing
7		Other Minerals Mining and Dressing
8		Food Processing
9		Food Production
10		Beverage Production
11		Tobacco Processing
12		Textile Industry
13		Garments and Other Fiber Products
14		Leather, Furs, Down and Related Products
15		Timber Processing, Bamboo, Cane, Palm and Straw Products
16		Furniture Manufacturing
17		Papermaking and Paper Products
18		Printing and Record Medium Reproduction
19		Cultural, Educational and Sports Articles
20		Petroleum Processing and Coking
21		Raw Chemical Materials and Chemical Products
22		Medical and Pharmaceutical Products
23		Chemical Fiber Products
24		Rubber Products
25		Plastic Products
26		Nonmetal Mineral Products
27		Smelting and Pressing of Ferrous Metals
28		Smelting and Pressing of Nonferrous Metals
29		Metal Products
30		Ordinary Machinery
31		Equipment for Special Purpose
32		Transportation Equipment Products
33		Electric Equipment and Machinery Products
34		Electronic and Telecommunications Equipment
35		Instruments, Meters, Cultural and Office Machinery Products
36		Other Manufacturing Industry
37		Scrap and Waste
38		Electric Power, Steam and Hot Water Production and Supply
39		Gas Production and Supply
40		Tap Water Production and Supply
41	Services	Transport, Storage, Postal and Telecommunication Services
42		Wholesale, Retail Trade and Catering Services
43		Other
44	Residents	Urban Residents
45		Rural Residents

### Constructing the high-resolution gridded database of Hg emissions in China

The atmospheric Hg emission inventories of each provincial administrative region and each sector in China were gridded using land-use data, individual enterprise data, road network data, and population data for China, as elaborated below. The data sources are presented in Table 2. The following is a brief description of the spatial processing of various sectors.

**Table 2:** Summary of data sources used to develop the gridded Hg emissions dataset.

Data Types	Data sources	Details
Land use dataset	Resource and Environment Science and Data Center, CAS^28^	China’s national land use and land cover change data at 1 km × 1 km grid resolution for the years 1995, 2000, 2005, 2010 and 2015
Enterprise data	Chinese Industrial Enterprises Database (CIED)^29^	Enterprise data by industry for the years 1998 to 2013
Road data	OpenStreetMap (OSM)^31^	China’s road network and traffic data
Population dataset	Resource and Environment Science and Data Center, CAS^34^	China’s population distribution data at 1 km × 1 km grid resolution for the years 1995, 2000, 2005, 2010 and 2015

#### Agriculture

High-resolution spatial gridding of agricultural atmospheric Hg emissions was performed using land-use data from China’s National Land Use and Cover Change (CNLUCC) dataset^28^ released by the Chinese Academy of Sciences (CAS). This study determines agricultural land use at a scale of 1 km × 1 km based on this dataset. The land use grid data is converted into agricultural land use data where plots of agricultural land are indicated by 1 (0 indicates other types of land). The CNLUCC data is available for 1995, 2000, 2005, 2010, and 2015. Data for the intermediate years were obtained through interpolation. The interpolation process is described below, taking the example of intermediate years between 2010 and 2015.

$$2011\;land\;use\;data=2010\;land\;use\;data+(2015\;land\;use\;data-2010\;land\;use\;data)\times 0.2$$

$$2012\;land\;use\;data=2010\;land\;use\;data+\left(2015\;land\;use\;data-2010\;land\;use\;data\right)\times 0.4$$

$$2013\;land\;use\;data=2010\;land\;use\;data+\left(2015\;land\;use\;data-2010\;land\;use\;data\right)\times 0.6$$

$$2014\;land\;use\;data=2010\;land\;use\;data+\left(2015\;land\;use\;data-2010\;land\;use\;data\right)\times 0.8$$

That is, if the land use type of a grid changed between 2010 and 2015 (from 1 to 0, for instance), the value in the grid in 2011 is 0.8, in 2012 is 0.6, in 2013 is 0.4, and 0.2 in 2014. Then, atmospheric Hg emissions in agricultural activities in each province are distributed into grids based on the value of each grid.

#### Industries

We used the Chinese Industrial Enterprises Database (CIED)^29^ to spatially distribute the atmospheric Hg emissions of industrial sectors. The database has been processed in our previous study, as there are inconsistencies in statistical caliber adjustment and industrial classifications^30^. The brief process procedure can be described as follows.

First, CIED covers the years from 1998 to 2013. Before 2011, the covered enterprises had annual revenue of more than 5 million yuan, while more than 20 million yuan since 2011. We constructed the enterprise data with annual revenues of over 5 million yuan from 1998 to 2014. On one hand, this study fills the data of 2011–2013 with enterprises with annual revenues of less than 20 million yuan in 2010. On the other hand, this study used the data of 2013 to spatially distribute the atmospheric Hg emissions of industrial sectors in 2014.

Second, the industrial classification in CIED for 1998–2001 goes with the Chinese industrial sector classification in 1994, whereas the 2002 version of classification is applied for CIED in 2002–2014. We unified the classification of CIED to the Chinese industrial sector classification in 2002, as it is similar to the CEADs database, which is used to convert Hg emissions from energy combustion sources to energy-using sectors. The only exception happens in the “Logging and Transport of Wood and Bamboo” sector of the CEADs database, which could not be directly matched to the classification in CIED. It is mapped into the “Farming, Forestry, Animal husbandry and Fishery” sector, and hence the Hg emissions of this sector are counted as agricultural emissions.

The spatial processing of atmospheric Hg emissions in industrial sectors was carried out in two steps. First, atmospheric Hg emissions of individual enterprises are estimated based on their total outputs. Then, the emissions of all the enterprises of one sector in one grid are added up to obtain the sector-wise Hg emissions of the grid.

#### Services and residents

In this category, Hg emissions in the transportation sector are spatialized with roadmaps, while those of other service sectors are distributed based on population.

For the transportation sector, we spatialized the aggregate emissions of this sector to each grid based on the traffic and road network data from OpenStreetMap (OSM)^31^. OSM is a free, open-source, and fast-developing map service jointly created by volunteers^32^. With years of development and increasing popularity, the quality of OSM data has been constantly improving and verified in some countries and regions. This study used the OSM road network data of 2015, the map with the highest quality in the research period, to calculate the emissions of the transportation sector each year. The widths of different types of routes in the road network were delineated according to the classification based on the width of roads in the Interim Provisions on Urban Planning Quota Index^33^. The routes were then converted into areas and gridded. The gridded routes are the proxy for spatial gridding of atmospheric Hg emissions from the transportation sector.

For other service sectors and residents, we used gridded population distribution data to proxy atmospheric Hg emissions. We distribute the atmospheric Hg emissions from the service sector and residential sector at the 1 km × 1 km spatial resolution based on China’s Population Spatial Distribution Kilometer Grid Dataset^34^. This dataset is available for the years 1995, 2000, 2005, 2010, and 2015. The values for the intermediate years were interpolated in the following manner, taking the years between 2010 and 2015 as an example.

$$Population\;in\;2011=Population\;in\;2010+\left(Population\;in\;2015-Population\;in\;2010\right)\times 0.2$$

$$Population\;in\;2012=Population\;in\;2010+\left(Population\;in\;2015-Population\;in\;2010\right)\times 0.4$$

$$Population\;in\;2013=Population\;in\;2010+\left(Population\;in\;2015-Population\;in\;2010\right)\times 0.6$$

$$Population\;in\;2014=Population\;in\;2010+\left(Population\;in\;2015-Population\;in\;2010\right)\times 0.8$$

China’s Population Spatial Distribution Kilometer Grid Dataset is gridded through a series of processing to the statistical population data of districts and counties, which lead to discrepancies. Because the total national population of China’s Population Spatial Distribution Kilometer Grid Dataset is not equal to the China Statistical Yearbook^35^, we calibrated the total population for each province based on the China Statistical Yearbook. We first calculated the total population of each province in the grid dataset and then estimated the calibration coefficients with the population of each province in the China Statistical Yearbook. The gridded population dataset is then calibrated with these coefficients to make it consistent with the total population as per the China Statistical Yearbook.

Moreover, the emissions from the category of Residents are divided into “urban residents” and “rural residents.” We further process the urban population based on the land type of “urban land,” while the rural population with “rural land” in CNLUCC. Finally, atmospheric Hg emissions of urban and rural residents in each province of China are distributed based on the gridded urban and rural population.

## Data Records

The database developed in this study provides a high-resolution (1 km × 1 km) spatial grid anthropogenic Hg emission dataset in China for the years 1998 to 2014. It is available from Zenodo^36^. The dataset includes 43 production sources and two household sources of atmospheric Hg emission in three forms (Hg0, HgII, and Hg_P_) for each year. The dataset values are in tons. Table 3 shows the naming protocol of the data files of the various sector categories (namely Agriculture, Industries, Services, Residents, For particular sectors, and Total).

**Table 3:** Nomenclature of data files for each of the sector categories in the database.

Sectors	File nomenclatures	Examples
Agriculture	form_1_year.tif	Hg0_1_1998.tif
Industries	form_ind_year.tif	Hg2_ind_2000.tif
Services	form_serv_year.tif	HgP_serv_2002.tif
Residents	form_resi_year.tif	HgP_resi_2005.tif
For particular sectors	form_number_year.tif	Hg0_2_2012.tif
Total	form_total_year.tif	Hg_total_2010.tif

**Fig. 2:**
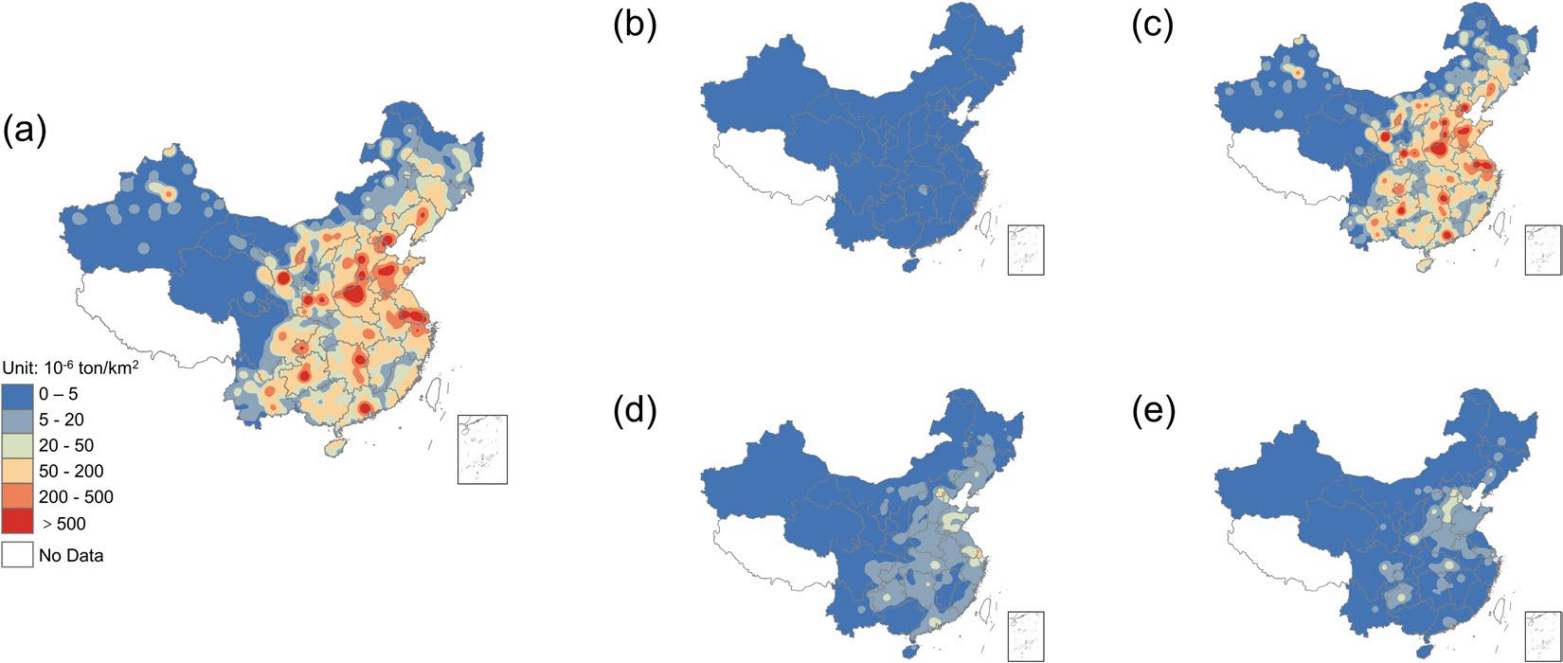
The kernel density maps of anthropogenic atmospheric Hg emissions from various sectors in China in 2014. (**a**) shows the total atmospheric Hg emissions in China. (**b**–**e**) show the atmospheric Hg emissions from China’s agriculture, industries, services, and residents, respectively.

Figure 2 shows the distribution of Chinese anthropogenic Hg emissions in 2014. We use a kernel density map to show the spatial distribution and characteristics of atmospheric Hg emissions in China to visualize hotspots and gradients. The kernel density model is a nonparametric method to estimate the probability density function of variables and is used to demonstrate the spatial distribution of relative emissions (rather than absolute values)^30,37^. The colors in the map represent the probability density of atmospheric Hg emissions calculated by the kernel density model after “aggregate” (cell factor is 10).

## Technical Validation

There are two sources of uncertainty in China’s high-resolution spatial gridded anthropogenic atmospheric Hg emissions dataset. On one hand, uncertainties are related to the accuracy of the data used; and on the other hand, uncertainties are associated with the calculation techniques such as data interpolation. First, there are some uncertainties in the CIED, such as incomplete data of enterprises at the micro level, position offset, or discrepancies between actual production emissions and statistical positions. Second, there are some uncertainties in the spatial grid land use data and population distribution when we carry out spatial processing. Third, in view of the discontinuous time series of spatially gridded land use data and population distribution data, this study uses interpolation to estimate the values for intermediate years. The values for those intermediate years, therefore, have some associated uncertainty.

The uncertainty of our dataset can be reduced by considering the following three aspects: 1) For the intermediate years calculated by the interpolation method, more accurate grid maps for land use and population distribution in the future can improve the accuracy of our dataset. 2) Future studies should improve the monitoring of Hg emission data and develop emission factors for specific production scales and production processes in specific regions. 3) For industrial enterprises, reliable bottom-up Hg emission inventories should be constructed in the future, which can be used to replace the data of the relevant sectors in this study. This requires reliable and publicly available bottom-up information for enterprises of the relevant sectors.

There are mainly two sources of errors in the newly prepared dataset. Firstly, in CIED, data for certain industries in some years and provinces are missing or incorrectly mapped, and enterprise information is incomplete, resulting in errors in the emissions value of the industrial sector. Secondly, errors can arise when dealing with inter-provincial boundaries or coastlines during provincial data validation using ArcGIS. For example, if a grid is located at the junction of Tianjin Province and Hebei Province, the Hg emissions in Hebei may be counted as the Hg emissions in Tianjin. If part of a grid is at the junction of sea and land, its Hg emissions may not be counted.

To assess the reliability of our dataset, we added up the Hg emissions of all the grids (for each year and province) and compared them with the inventory of Wu *et al*.^11,12^. The marginal errors of total Hg emissions in most industrial sectors were less than 5%. The industry sector has the highest error percentage associated with it. The data of agricultural emissions match 100% except for Ningxia in 2000 and 2001, which has an error of 0.02%; the error of industrial emissions is less than 2.57%; the error of service sector emissions is less than 0.57%; the error of residential emissions is less than 1.18%. The top 10 years and provinces of total errors are shown in Table 4. The top 10 years and provinces of industrial errors are shown in Table 5.

**Table 4:** Top 10 years and provinces ranked by total atmospheric Hg emission errors.

Ranking	Years	Provinces	Hg emission errors				
			Agriculture	Industries	Services	Residents	Total
1	2014	Liaoning	0.00% (0.0000)	2.47% (−0.3714)	0.01% (−0.0001)	0.18% (−0.0014)	2.13% (−0.3729)
2	2002	Fujian	0.00% (0.0000)	2.57% (−0.0886)	0.04% (−0.0002)	0.44% (−0.0012)	2.12% (−0.0900)
3	2004	Shandong	0.00% (0.0000)	2.04% (−0.6479)	0.02% (0.0001)	0.10% (−0.0006)	1.94% (−0.6484)
4	2013	Liaoning	0.00% (0.0000)	1.95% (−0.3110)	0.02% (0.0002)	0.19% (−0.0013)	1.71% (−0.3121)
5	2005	Shandong	0.00% (0.0000)	1.66% (−0.7003)	0.00% (0.0000)	0.15% (−0.0015)	1.56% (−0.7018)
6	2006	Shandong	0.00% (0.0000)	1.51% (−0.6596)	0.01% (−0.0002)	0.13% (−0.0013)	1.42% (−0.6612)
7	2002	Shanxi	0.00% (0.0000)	1.52% (−0.1342)	0.02% (−0.0001)	0.06% (−0.0004)	1.32% (−0.1347)
8	2012	Liaoning	0.00% (0.0000)	1.48% (−0.2278)	0.02% (0.0003)	0.20% (−0.0016)	1.28% (−0.2291)
9	2001	Shanxi	0.00% (0.0000)	1.47% (−0.1121)	0.02% (−0.0001)	0.06% (−0.0004)	1.26% (−0.1126)
10	2007	Shandong	0.00% (0.0000)	1.32% (−0.5636)	0.03% (−0.0005)	0.16% (−0.0016)	1.24% (−0.5657)

The specific values of the errors are listed in parentheses (unit: tons).

**Table 5:** Top 10 years and provinces ranked by industrial atmospheric Hg emission errors.

Ranking	Years	Provinces	Hg emission errors				
			Agriculture	Industries	Services	Residents	Total
1	2002	Fujian	0.00% (0.0000)	2.57% (−0.0886)	0.04% (−0.0002)	0.44% (−0.0012)	2.12% (−0.0900)
2	2014	Liaoning	0.00% (0.0000)	2.47% (−0.3714)	0.01% (−0.0001)	0.18% (−0.0014)	2.13% (−0.3729)
3	2004	Shandong	0.00% (0.0000)	2.04% (−0.6479)	0.02% (0.0001)	0.10% (−0.0006)	1.94% (−0.6484)
4	2013	Liaoning	0.00% (0.0000)	1.95% (−0.3110)	0.02% (0.0002)	0.19% (−0.0013)	1.71% (−0.3121)
5	2005	Shandong	0.00% (0.0000)	1.66% (−0.7003)	0.00% (0.0000)	0.15% (−0.0015)	1.56% (−0.7018)
6	2002	Shanxi	0.00% (0.0000)	1.52% (−0.1342)	0.02% (−0.0001)	0.06% (−0.0004)	1.32% (−0.1347)
7	2006	Shandong	0.00% (0.0000)	1.51% (−0.6596)	0.01% (−0.0002)	0.13% (−0.0013)	1.42% (−0.6612)
8	2012	Liaoning	0.00% (0.0000)	1.48% (−0.2278)	0.02% (0.0003)	0.20% (−0.0016)	1.28% (−0.2291)
9	2001	Shanxi	0.00% (0.0000)	1.47% (−0.1121)	0.02% (−0.0001)	0.06% (−0.0004)	1.26% (−0.1126)
10	2002	Hainan	0.00% (0.0000)	1.41% (−0.0047)	0.04% (−0.00001)	0.32% (−0.0001)	1.20% (−0.0048)

The specific values of the errors are listed in parentheses (unit: tons).

## Supplementary information

The matching relationship matrix

## Data Availability

The produced datasets elaborated in this work were constructed based on custom-built codes written in Matlab and ArcGIS 10.2. The codes and 1 km×1 km Chinese anthropogenic Hg emission data^36^ can be obtained from zenodo or by contacting the designated staff at https://cgeed.net.
